# Prognostic interplay between cardiac allograft vasculopathy and coronary vascular function by hybrid rubidium-82 PET/CT imaging in heart transplant population

**DOI:** 10.1007/s00259-025-07676-3

**Published:** 2025-11-26

**Authors:** Roberta Assante, Emilia Zampella, Irene Mattucci, Adriana D’Antonio, Francesco Curcio, Teresa Mannarino, Pietro Buongiorno, Mariarosaria Panico, Cristiano Amarelli, Paolo Golino, Pasquale Abete, Francesco Cacciatore, Alberto Cuocolo, Wanda Acampa

**Affiliations:** 1 Department of Advanced Biomedical Sciences, University of Naples Federico II, Via Pansini 5, Naples, 80131 Italy; 2 Department of Cardiac Surgery and Transplants, Monaldi, A.O. dei Colli, Naples, Italy; 3 Department of Translational Medical Sciences, University of Naples Federico II, Naples, Italy; 4 Institute of Biostructure and Bioimaging, National Council of Research, Naples, Italy

**Keywords:** Myocardial blood flow, Myocardial perfusion reserve, Coronary artery calcium, PET/CT, Heart transplant

## Abstract

**Purpose:**

We evaluated the interrelation of cardiac allograft vasculopathy (CAV) and both coronary artery calcium and coronary vascular function, as assessed rubidium-82 (^82^Rb) positron emission tomography (PET)/computed tomography (CT) imaging, in heart transplant population with respect to prediction of clinical outcomes.

**Methods:**

A total of 100 (mean age 60 ± 13 years) consecutive patients were studied. CAC score was measured according to the Agatston method and patients were categorized into 2 groups (< 100, and ≥100). Baseline and hyperemic MBF were automatically quantified. MPR was calculated as the ratio of hyperemic to baseline MBF and it was considered reduced when < 2.

**Results:**

During the mean time of 27 ± 8 months, 35 events occurred. Patients with events showed a higher prevalence of CAV, MPR impairment and CAC score > 100 as compared to patients without events. At multivariable COX analysis, CAC score, CAV and reduced MPR were independent predictors of events. In patients without previous CAV, the presence of reduced MPR was associated with higher event rate compared to normal MPR.

**Conclusions:**

In heart transplanted patients, the presence of CAV and reduced MPR were associated with a poor prognosis and higher risk of adverse events. In patients without CAV the presence of reduced MPR was associated with worst prognosis. Thus, the early noninvasive evaluation of microcirculatory dysfunction in HT patients has important clinical implication, providing a better risk stratification and subsequent modification of treatment strategies, with a potential impact on patient management at follow-up.

**Clinical trial number**: not applicable

## Introduction

Heart transplantation (HT) represents well-established curative treatment to improve survival in patients with end-stage heart failure [1]. Several studies have been published on risk factors, potential complications and therapy options in the early phase following HT. Advances in immunosuppression, surgical techniques and patient’s management have led to a significant decline of acute allograft rejection and improved survival after HT over time [2]. However, cardiac allograft vasculopathy (CAV) or acute cellular rejection remains a leading cause of mortality and morbidity in HT patients [3]. Myocyte damage and structural alteration of microvasculature are common phenotypes of acute allograft rejection. The coronary pathology in HT patients is based on a different etiology than the atherosclerosis in the general population and micro vasculopathy is associated with an increased risk of all-cause death and fatal cardiac event, dependent, but also independent of epicardial CAV [4]. Non-invasive cardiac imaging modalities have been used for the evaluation of HT patients [5, 6]. In particular, advanced echocardiography, cardiac magnetic resonance and coronary computed tomography angiography have been used to evaluate different aspects of the cardiac morphology and function [5]. However, these techniques were not useful to detect non angiographic but prognostically significant epicardial intimal and microvascular diseases [2].

Myocardial perfusion reserve (MPR) measurement by positron emission tomography (PET)/computed tomography (CT) represents the most validated noninvasive index of coronary vasodilator function, providing an accurate combined evaluation of both epicardial and microvascular compartments [6–9]. The associations between microvascular dysfunction or epicardial evaluation and adverse outcomes in HT patients have been evaluated separately [10, 11]. The combined overall evaluation of different parameters obtained by PET/CT would provide additional information for better risk stratification. The aim of the study was to evaluate the prognostic value of coronary vascular function and epicardial burden by hybrid rubidium-82 (^82^Rb) PET/CT imaging in HT population.

## Methods

### Patients

We prospectively studied 100 HT patients referred to rest/stress ^82^Rb PET/CT imaging for perfusion and functional evaluation between April 2022 and January 2023. We included patients with at least one invasive angiography or CT angiography during their routine CAV screening before PET/CT studies. Patients with revascularization procedures, myocardial infarction, or acute coronary syndrome between angiography/CCTA and PET imaging were excluded. At invasive coronary angiography CAV was defined according to International Society for Heart and Lung Transplantation nomenclature [12]. Any patient with ISHLT grade 1 or higher was considered to be abnormal and designated in a group with CAV. At IVUS images maximal intimal thickness (MIT) was determined, and MIT ≥ 0.5 mm was used to define CAV.

For each patient the presence of coronary risk factors was noted. Hypertension was defined as a blood pressure ≥140/90 mm Hg or the use of anti-hypertensive medication. Hypercholesterolemia was defined as total cholesterol level ≥6.2 mmol/L or treatment with cholesterol lowering medication. Patients were classified as having diabetes if blood glucose was greater than 126 mg/dl on two determinations or HbA1c greater than 6.5% or they were receiving treatment with oral hypoglycemic drugs or insulin. A positive family history of CAD was defined by the presence of disease in first-degree relatives younger than 55 years in men or 65 years in women. The review committee of our institution approved this study, and all patients gave informed consent (“Comitato Etico, Università Federico II”, protocol number 110/17).

### PET imaging

As a routine preparation, before PET/CT imaging, patients were asked to discontinue nitrates for 6 h, calcium channel blockers and caffeine-containing beverages for 24 h, and beta-blockers for 48 h. Rest and stress gated cardiac PET/CT images were acquired using Ingenuity TF scanner (GE Healthcare) as follows: after a CT scout to check patient position, a low-dose CT (0.4 mSv; 120 kVp; effective tube current, 26 mA [11-mAs quality reference]; 3.3 s) was performed for attenuation correction and CAC score measurement, during normal breathing before and after PET acquisitions. For both rest and stress imaging, 1110 MBq of ^82^Rb was injected intravenously with a 6-min list-mode PET acquisition. Dynamic PET acquisition was started at rest followed by pharmacologic stress test using Regadenoson (400 mcg intravenously over a 10-seconds period). Both rest and stress dynamic images were reconstructed into 26-time frames (12 × 5 s, 6 × 10 s, 4 × 20 s, and 4 × 40 s) using the vendor standard ordered subsets expectation maximization 3D reconstruction (2 iterations, 24 subsets) with 6.5-mm Gaussian postprocessing filter. The images were corrected for attenuation using the low-dose CT. Hemodynamic parameters and 12-lead ECG were recorded at baseline and throughout the infusion of adenosine.

### Imaging analysis

Trans-axial PET perfusion images were automatically reoriented into short-axis and vertical and horizontal long-axis slices. Myocardial perfusion was assessed using standardized segmentation of 17 myocardial regions using automated software (Cedars-Sinai Medical Center, Los Angeles, California) [13]. Left ventricular ejection fraction (LVEF) has been also evaluated by gated acquisitions. The total perfusion defect (TPD) was considered normal when < 5% of the total left ventricle [14]. Myocardial blood flow (MBF) was calculated (mL/min/g) from the dynamic rest and stress imaging series with commercially available software (FlowQuant, University of Ottawa Heart Institute) [15]. From the ratio of hyperemic to baseline MBF, MPR was calculated and considered reduced when < 2. Coronary calcification was defined as a plaque with an area of 1.03 mm2 and a density ≥ 130 HU. The CAC score measurements were estimated according to the method described by Agatston et al. [16]. CAC scores were also categorized into two groups, < 100 and ≥ 100 [17]. Experienced nuclear medicine physicians analyzed the CT studies, blinded to the PET results. Epicardial adipose tissue (EAT) density was quantified on unenhanced CT images a commercially available platform (Osirix v. 4.1.2, Bernex, Switzerland). The image processing started at the level of the pulmonary trunk and ended at the level of the inferior diaphragmatic surface of the heart to manually trace pericardial borders. The area outside the traced pericardium was excluded. The range of attenuation for EAT segmentation was then set between − 30 and − 190 HU excluding myocardium, coronary arteries, coronary calcium, the aorta, and blood pool [18, 19].

### Outcomes

Follow-up was obtained by using a questionnaire that was assessed by a phone call to all patients or referring physicians and by reviewing hospital or physicians’ records. The outcome endpoints considered were composite of all-cause death, acute cellular rejection or heart failure hospitalization, whichever occurred first. The death was confirmed by review of death certificate, hospital chart, or physician’s records. The date of the last examination or consultation was used to determine the length of follow-up.

### Statistical analysis

Continuous data were expressed as median (interquartile range) for the non-normally distributed continuous variables and compared using Mann-Whitney U test. Categorical variables were expressed as number (%) and compared for the differences by χ2 and Fisher’s exact test as appropriate. The ln(CAC score + 1) transformation was used to adjust for the rightward skew of the data and to reduce heteroscedasticity. CAC score was then categorized into 2 groups, < 100 and ≥ 100. Relationships among variables were evaluated using Spearman’s rank correlation analysis. A P value < 0.05 (two-sided) was considered statistically significant. Annualized event rate, expressed as % person-years, was calculated as the cumulative number of events divided by person-time. Hazard ratios with 95% confidence intervals were calculated by univariable and multivariable Cox regression analysis. Variables showing a *P* value < 0.05 at univariable analysis were considered for multivariable analysis. To assess the robustness of the multivariable Cox regression model, a bootstrap analysis with 1000 resampled datasets (sampling with replacement) was performed. Event-free survival curves were obtained by the Kaplan-Meier method and compared using the log-rank test. A parametric survival model was used to identify how the variables influenced time to event and to estimate cumulative hazard during the follow-up. Statistical analysis was performed with Stata 18 software (StataCorp, College Station, Texas USA).

## Results

### Patient characteristics

A total of 100 patients underwent rest–stress Rb-82 PET/CT between April 2022 and January 2023. The mean time between HT and cardiac imaging was 154 *±* 69 months. Of the overall patient population, 26 (26%) performed CT angiography and 74 (74%) submitted to invasive angiography. The mean time between ICA or CCTA and the PET/CT scan was 24 + 10 months. The baseline characteristics and imaging findings in the overall population are shown in Tables 1 and 2. Thirty-two patients (32%) had a diagnosis of CAV before PET imaging on invasive angiography or CT angiography. Twenty (62%) of the 32 patients with CAV showed a reduced MPR as compared to 12 (18%) of the 66 patients without CAV (*P* <.001). Patients with CAV showed lower values of hyperemic MBF and MPR as compared to patients without CAV (2.31 ± 0.73 vs. 3.38 ± 1.09 and 1.93 ± 0.83 vs. 2.60 ± 0.84, respectively, both *P* <.001). Differently, baseline MBF were similar in patients with and without CAV. Among the 20 patients with CAV and reduced MPR, in 12 (60%) patients the MPR reduction was related to increase in resting MBF (endogen/functional microvascular disease) and in 8 (40%) patients the MPR reduction was related to decrease in hyperemic MBF (classical/structural microvascular).

**Table 1:** Baseline clinical characteristics of patient population according to events

	All patients *(n* = 100)	Events *(n* = 35)	No events *(n* = 65)	*P* value
Age (years)	60 ± 12	63 ± 13	59 ± 12	0.13
Male gender, *n* (%)	73 (73)	26 (74)	47 (72)	0.83
Diabetes, *n* (%)	37 (37)	17 (48)	20 (31)	0.08
Hypertension, *n* (%)	71 (71)	22 (63)	49 (75)	0.18
Dyslipidemia, *n* (%)	68 (68)	25 (71)	43 (66)	0.59
Smoking history, *n* (%)	49 (49)	22 (63)	27 (41)	< 0.05
Family history of CAD, *n* (%)	38 (38)	14 (40)	24 (37)	0.76
Previous documented CAV, *n* (%)	32 (32)	22 (63)	10 (15)	< 0.001
Immune-suppressant medications				
Cyclosporine, *n* (%)	70 (70)	29 (83)	41 (63)	< 0.05
Tacrolimus, *n* (%)	30 (30)	6 (17)	24 (37)	< 0.05
Everolimus, *n* (%)	50 (50)	21 (60)	29 (45)	0.14
Cortisone, *n* (%)	60 (60)	18 (51)	42 (65)	0.20
Other medications				
Statins, *n* (%)	70 (70)	26 (74)	44 (68)	0.49
Ivabradine, *n* (%)	12 (12)	8 (23)	4 (6)	< 0.05
Metformin, *n* (%)	12 (12)	3 (8)	9 (14)	0.43

Values are expressed as mean value ± standard deviation or as number (percentage) of subjects. *CAD* coronary artery disease; *CAV* coronary allograft vasculopathy

**Table 2:** Imaging findings of patient population according to events

	All patients *(n* = 100)	Events *(n* = 35)	No events *(n* = 65)	*P* value
Baseline MBF [mL/min/g]	1.54 ± 0.4	1.48 ± 0.3	1.57 ± 0.5	0.38
Hyperemic MBF [mL/min/g]	3.03 ± 1.1	2.42 ± 0.9	3.37 ± 1.0	< 0.001
MPR	2.39 ± 0.8	1.91 ± 0.8	2.63 ± 0.8	< 0.001
MPR < 2, n [%]	34 (34)	20 (57)	14 (21)	< 0.001
Ln (CAC + 1) score	1.48 ± 2.4	2.88 ± 2.9	0.73 ± 1.7	< 0.001
CAC score < 100	80 (80%)	21 (60%)	59 (91%)	< 0.001
CAC score ≥ 100	20 (20%)	14 (40%)	6 (9%)	< 0.001
Abnormal MPI	18 (18)	9 (26)	9 (14)	0.14
LVEF	66 ± 10	63 ± 14	68 ± 8	< 0.05
Mean EAT density	−79.6 ± 3.7	−79.7 ± 3.8	−79.6 ± 3.7	0.89

Values are expressed as mean value ± standard deviation or as number (percentage) of subjects

*MBF* myocardial blood flow; *MPR* myocardial perfusion reserve; *CAC* coronary artery calcium; *MPI* myocardial perfusion imaging; *LV*EF left ventricular ejection fraction; *EAT* epicardial adipose tissue

**Fig. 1:**
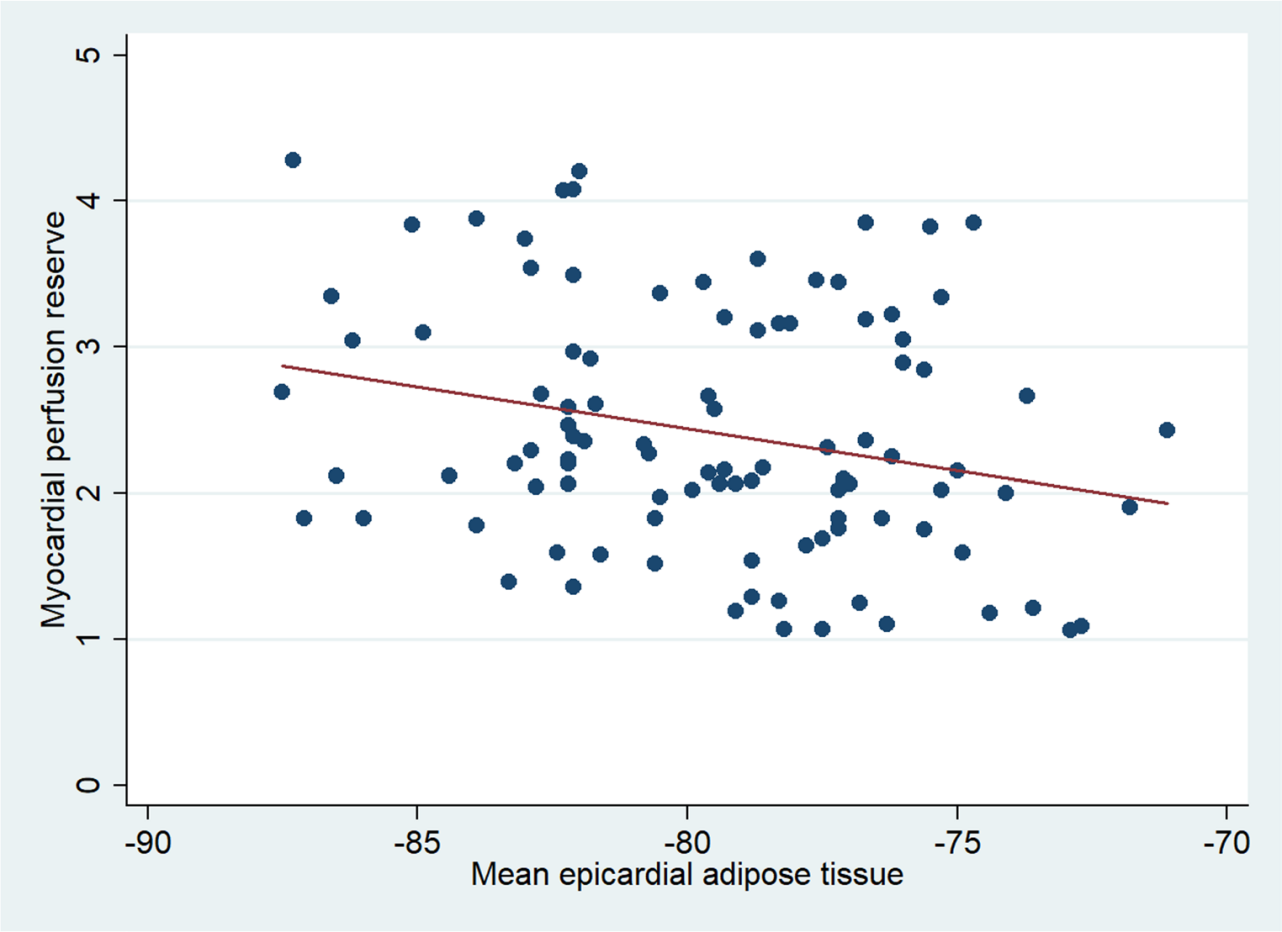
Correlation between myocardial perfusion reserve (MPR) versus mean epicardial adipose tissue (EAT) volume

Interestingly, patients with CAV showed lower values of mean EAT density as compared to those without CAV (−81.1 ± 3.7 vs. −78.9 ± 3.5, *P* <.01). A significant inverse relationship between MPR and mean EAT density has been demonstrated (*r* = -.25, *P* <.05) (Fig. 1). Similarly, a significant correlation was found between MPR and Ln (CAC + 1) score (*r* = -.21, *P* >.05). Differently, no significant correlation between Ln (CAC + 1) score and mean EAT density was observed (*P* =.07).

### Patient outcomes

Follow-up was available in the overall 100 patients included in the study. During a mean time of 27 ± 8 months from PET imaging, 35 events occurred (35% cumulative event rate, with an annual event rate of 13% person-years). The events were death in 13 (37%) patients, acute cellular rejection in 5 (14%) patients and heart failure hospitalization in 17 (48%) patients. As shown in Table 1, patients with events had a higher prevalence of CAV compared to patients without events. All the other baseline clinical characteristics were comparable between the two groups. Moreover, patients with events showed lower hyperemic MBF and MPR values, lower values of resting LVEF, a higher prevalence of MPR impairment and CAC score > 100 as compared to patients without events (Table 2).

### Predictors of events

AER was significantly higher in patients with CAV as compared to those without CAV (31% vs. 6.5%, *P* <.001). Similarly, patients with reduced MPR showed higher AER as compared to those with normal MPR (28% vs. 7.5%, *P* <.001).

**Fig. 2:**
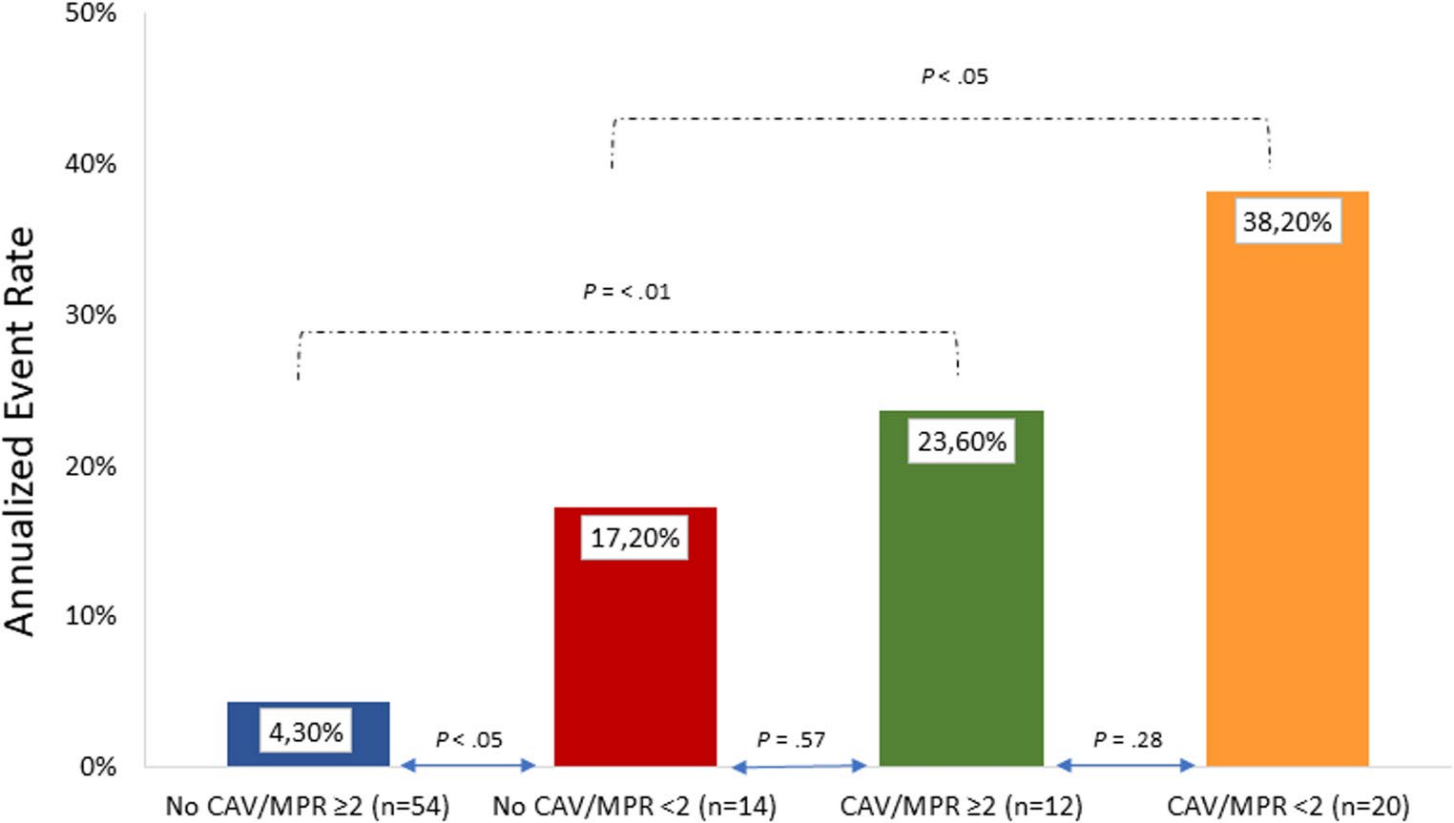
Annualized event rate according to normal or reduced myocardial perfusion reserve (MPR) and absence or presence of cardiac allograft vasculopathy (CAV)

**Fig. 3:**
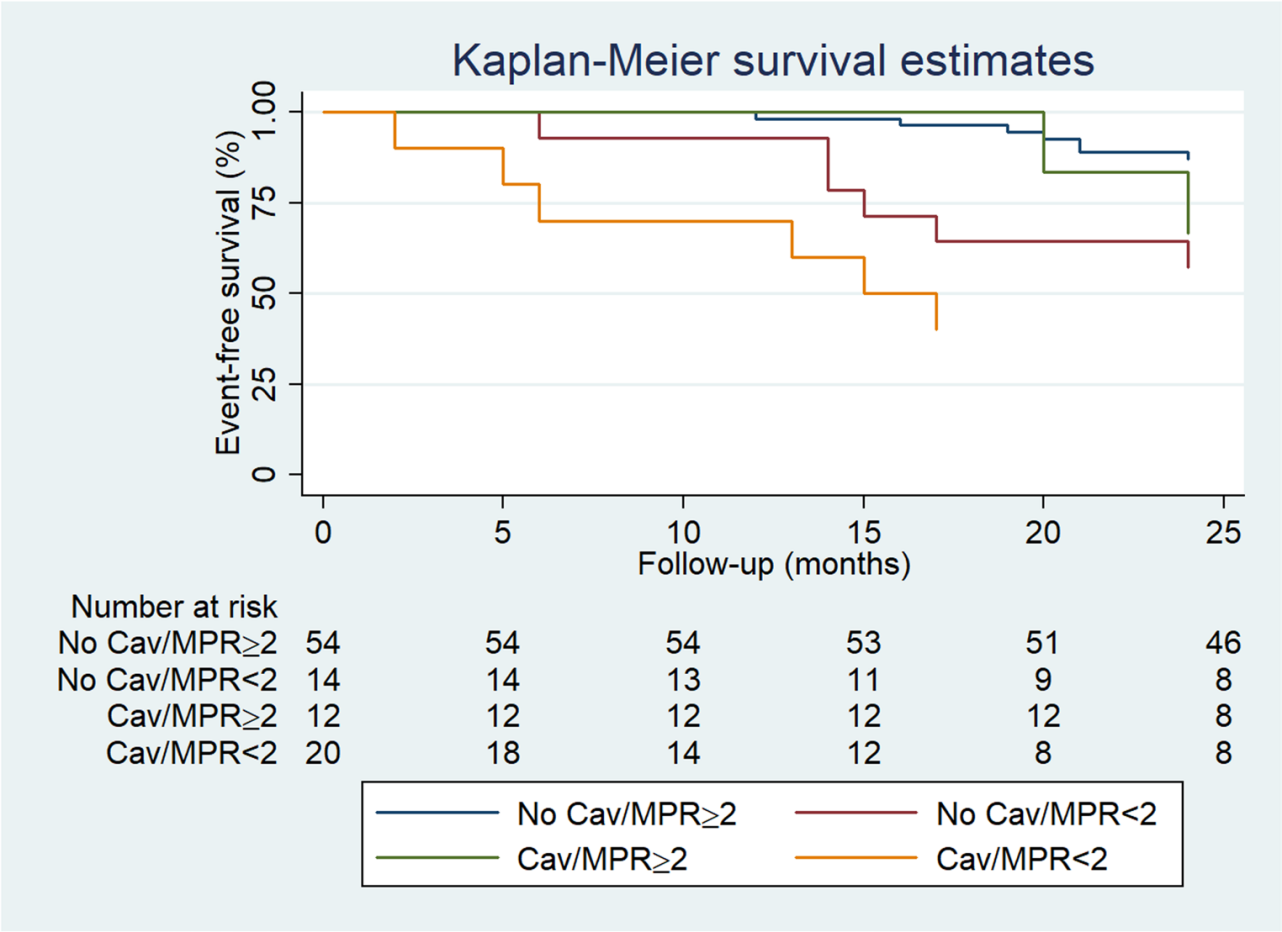
Event-free survival curves by Kaplan-Meyer analysis according to myocardial perfusion reserve (MPR) and cardiac allograft vasculopathy (CAV)

AER according to CAV and MPR are depicted in Fig. 2. As shown, reduced MPR in patients with CAV was associated with highest event rate. However, in patients with CAV no significant differences were observed in AER, in the presence or absence of reduced MPR. Interestingly, patients without CAV showed higher AER in the presence of reduced MPR as compared to those with preserved MPR (*P* = < 0.05). Moreover, the AER of the patients with endogen microvascular disease (*n* = 12) and classical microvascular disease (*n* = 8) was similar (31% vs. 42%, *P* =.61). The event-free survival curves according to CAV and MPR are reported in Fig. 3. As shown, patients with CAV and reduced MPR showed the worst outcome. Moreover, event-free survival was lower in patients with no CAV and reduced MPR as compared to those with no CAV and normal MPR (*P* <.05). Univariable Cox regression analyses are reported in Table 3. CAC score, CAV, reduced MPR and LVEF were univariable predictors of events. At multivariable analysis, CAC score (*P* <.01), the presence of CAV and reduced MPR (both *P* <.05) were independent predictors of events. The bootstrap derived HR were consistent with the original estimates and the statistical significance of CAC score, the presence of CAV and reduced MPR was maintained. The bootstrap bias for CAC score, the presence of CAV and reduced MPR was 0.01, 0.004 and 0.04, respectively.

**Table 3:** Univariable and multivariable predictors of adverse events

	Univariable analysis		Multivariable analysis	
	HR (95% CI)	*P*-value	HR (95% CI)	*P*-value
Age	1.02 (0.99–1.05)	0.12		
Male gender	1.04 (0.48–2.20)	0.91		
Diabetes	1.83 (0.94–3.56)	0.07		
Hypertension	0.62 (0.31–1.24)	0.18		
Dyslipidemia	1.18 (0.57–2.47)	0.65		
Smoking history	1.98 (0.99–3.94)	0.05		
Family history of CAD	1.16 (0.59–2.28)	0.66		
Previous CAV	4.87 (2.45–9.70)	< 0.001	2.72 (1.26–5.87)	< 0.05
Ln (CAC + 1) score	1.25 (1.12–1.41)	< 0.001	1.18 (1.04–1.33)	< 0.01
MPR < 2	3.96 (2.02–7.77)	< 0.001	2.57 (1.24–5.32)	< 0.05
LVEF	0.95 (0.92–0.98)	< 0.005		
Mean EAT density	1.00 (0.91–1.10)	0.88		

*HR* hazard ratio; *CI* confidence interval; *CAD* coronary artery disease; *CAC* coronary artery calcium; *CAV* coronary allograft vasculopathy; *MPR* myocardial perfusion reserve; *LV*EF left ventricular ejection fraction; *EAT* epicardial adipose tissue

### Change in risk with time

**Fig. 4:**
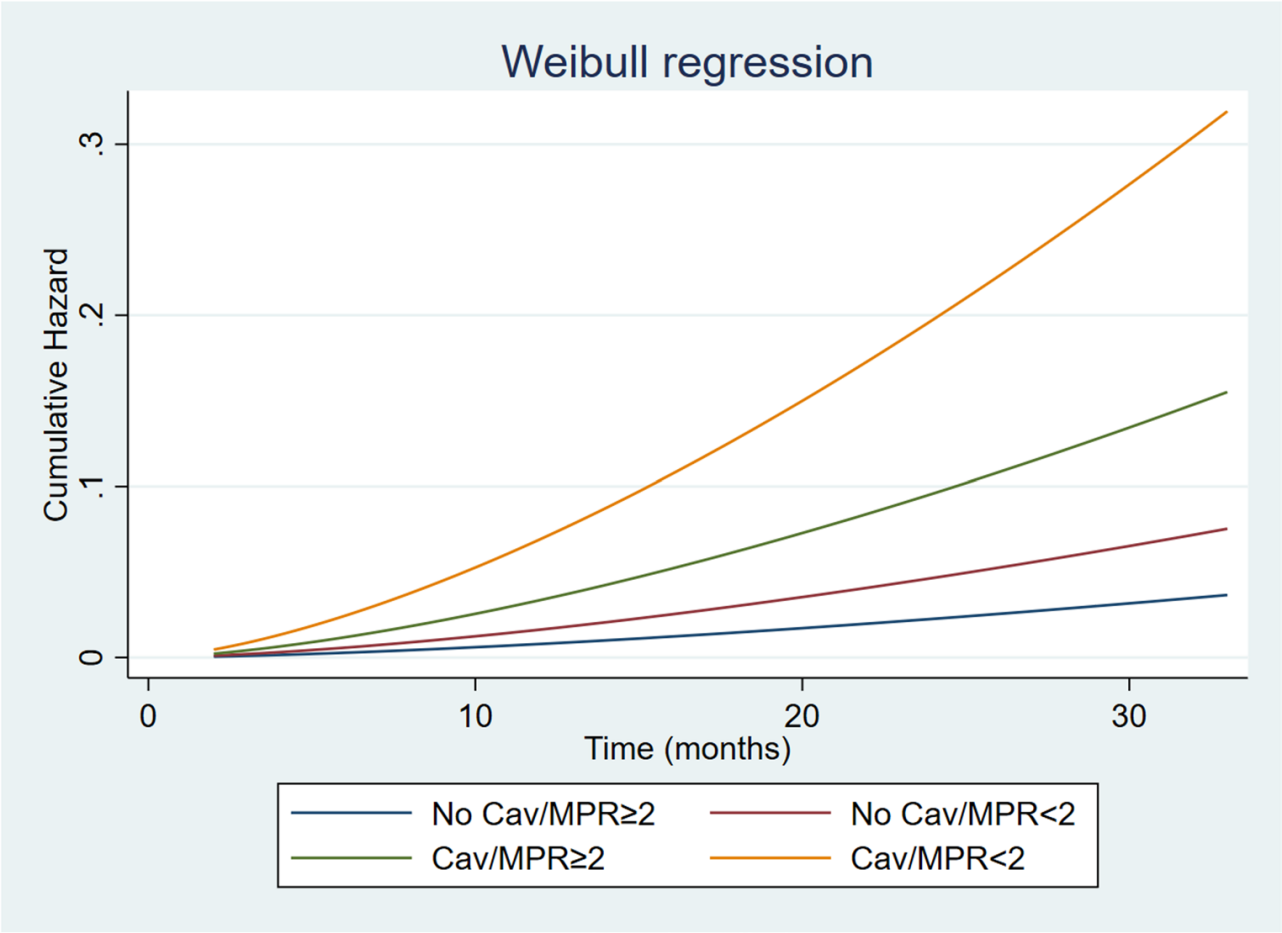
Estimated probability of events in cardiac allograft vasculopathy (CAV) and no CAV patients stratified by myocardial perfusion reserve (MPR)

The predicted cumulative hazard according to CAV and MPR status is depicted in Fig. 4. Parametric survival analysis including in the model CAV and MPR revealed that the highest risk of cardiac events and the major acceleration over time was observed in CAV patients with reduced MPR. Interestingly, in patients with CAV and MPR ≥ 2, a lower risk acceleration over time as compared to patients with CAV and MPR < 2 was observed (Fig. 4).

### Separate analysis in patients with CAV and normal perfusion imaging

Among the overall HT patients with normal myocardial perfusion (*n* = 82), patients with CAV (*n* = 22) showed lower values of hyperemic MBF and MPR as compared to patients without CAV (*n* = 60) (2.47 ± 0.68 vs. 3.43 ± 1.09 and 2.09 ± 0.93 vs. 2.68 ± 0.84, respectively, both *P* <.01). Moreover, similarly to the overall patient population, reduced MPR in patients with CAV was associated with highest event rate (41%). In patients with CAV no significant differences were observed in AER, in the presence or absence of reduced MPR (21% vs. 41%, *P =*.21*)*. Interestingly, patients without CAV showed higher AER in the presence of reduced MPR as compared to those with preserved MPR (24% vs. 4%, *P* = < 0.005).

## Discussion

The present study demonstrated that in HT patients both the presence of CAV and reduced MPR are associated with increased and accelerated risk of adverse events after about three years of follow-up. HT patients with CAV and reduced MPR showed the highest event rate, with poor event-free survival. Interestingly, also patients without CAV and reduced MPR showed higher event rate compared to those with normal MPR.

Despite the advances in pharmacological therapy and patient management, graft dysfunction including both acute allograft rejection (AAR) and CAV remain the leading cause of morbidity and mortality in HT patients. AAR-related deaths account for 11% of all deaths in the first three years following transplantation [4]. The incidence of CAV, affecting all epicardial allograft arteries, concerns approximately 30% of patients five years after transplantation and 50% of patients ten years after transplantation [20]. Cardiac graft dysfunction represents a distinct form of disease that is the most important long-term complication of heart transplantation, with a complex pathophysiology not yet fully investigated. Coronary microcirculation disease (CMD) affected before any clinical manifestations of the rejection process seems to be the common factor in the pathogenesis of both AAR and CAV. Thus, for HT patients, establishing early predictors of graft dysfunction may be crucial for patients’ management and short and long-term prognosis. Quantitative PET/CT provides a noninvasive functional and physiological assessment of both the epicardial coronary arteries and the microvasculature with quantification of different parameters such as perfusion, CAC score, MBF and MPR, demonstrating a high diagnostic accuracy and prognostic value in different categories of patients [21–24]. Multiple recent studies have shown that PET/CT with MBF quantification has diagnostic and prognostic utility for CAV identification and in predicting all-cause of mortality [25–27]. To detect epicardial CAV, PET/CT provided information on the health of the microvascular system, providing an important advantage over traditional invasive methods. In a previous study Bravo et al. demonstrated that quantifying absolute MBF using PET improves CAV detection compared to conventional semi-quantitative perfusion parameters, providing better patient stratification [25]. In this study, the authors developed a multiparametric non-invasive PET score including myocardial perfusion, absolute MBF quantification and allograft function and showing an important stepwise increased risk with worsening PET CAV score [25]. Konerman et al. found in 117 HT patients followed for median of 1.4 years found that, MPR resulted an independent predictor of the composite outcome of cardiovascular death, acute coronary syndrome, revascularization, or heart failure admission [26]. Moreover, different studies report that microcirculatory dysfunction can predict CAV and, when assessed regularly, can prevent graft dysfunction’s progression and improve patients’ survival after HTx [27–29].

Accordingly, in our study, HT patients with CAV showed a significantly reduced MPR as compared to patients without CAV, showing the worst outcome compared to the other categories of patients. Moreover, from our data it emerged that in the subgroup of patients without CAV, a reduced MPR was associated with a lower survival as compared to a preserved MPR in a follow-up of 3 years. Thus, reduced MPR is related to the presence of CAV, as already demonstrated, but could also be considered as an early sign of graft vasculopathy and/or events. Recently, HT patients with no history of CAV and normal myocardial perfusion, an endogen/functional pattern of CMD, characterized by high resting flows, was associated with higher rate of adverse events and death [30]. Classical/structural CMD, defined as poor augmentation of stress flow, was not associated with poor outcomes [29] in a subgroup of few patients and events. In our study, in the subgroup of patients without CAD, 43% of patients with abnormal MPR experienced events as compared to the 13% with normal MPR (*p* <.05).

Chronic inflammation plays a significant role in the development of complications after HT, including graft rejection and CAV. EAT is located between the myocardium and the visceral pericardium having local effects on myocardial microcirculation and for that is considered a clinical biomarker of cardiometabolic diseases. Considering the possibility to evaluate both anatomic and functional data by PET/CT study we tested also EAT value in this specific patient population.

Interestingly, from our data it emerged a significant inverse relationship between EAT and MPR. Published data demonstrated a significant impact of EAT density on chronic inflammation and atherosclerotic calcification probably through the mechanism of endocrine or paracrine [31–33]. In particular, lower EAT density was reported to be related with an adverse metabolic profile, independent of EAT volume [31]. Moreover, several studies have focused on the relationship between EAT and coronary vascular function [19, 34, 35]. In patients without overt CAD and normal myocardial perfusion imaging, EAT volume resulted associated with hyperemic MBF and MPR, confirming the influence of EAT also on microcirculation. Moreover, it has been suggested that EAT could promote endothelial dysfunction, and impaired MBF could aggravate adipose tissue hypoxia triggering a vicious circle. From this study the relationship between EAT and MPR is relatively weak. Moreover, EAT did not result significant in patient prognostication. However, the relation between EAT and MPR needs more attention to evaluate the possible role of EAT in the inflammation process and/or in the development of microvascular dysfunction. Thus, further investigations in large patient populations are necessary to evaluate the better identify EAT as marker of the early identification of coronary vascular dysfunction. Accordingly, the early assessment of the coronary microcirculation and other parameters obtained by PET/CT, including the EAT, could be useful to better understand the long-term cardiac complications after HT.

Published data evaluating the prognostic value of serial PET scans in HT patients demonstrated that the longitudinal progression of CAV is accompanied by a progressive decline of MPR [28]. Moreover, the natural progression of CAV after a low-risk PET/CT, as part of an annual screening program, demonstrated to have a role in guiding clinicians on longitudinally utilize PET/CT [29]. In our population the parametric survival analysis showed that patients with CAV and normal MPR showed a lower risk acceleration over time as compared to patients with CAV and reduced MPR, confirming that also in the presence of CAV, microvascular dysfunction contributes to accelerating the probability of adverse events. Thus, MPR by PET/CT could be used to evaluate the HT patients re-test time in a short and long-term follow-up. From a pathophysiologic point of view, a reduced MPR can be the result of the combination of different alterations such as impaired vasodilation, enhanced vasoconstrictor responsiveness and/or structural remodeling of the coronary microvasculature. Thus, the full understanding of the mechanism linking CAV and MPR in HT patients deserve further investigations, in particular the clinical significance of the potential results and how to use them to improve the prognosis of post-transplant patients in a long-term follow-up. Thus, the relationship between graft vasculopathy and MPR as index of microvascular disease should be better investigated in larger patients’ selection of patients with a without CAV. Moreover, it should be considered that cardiac allograft vasculopathy represents a distinct entity as compared to traditional CAD and conventional coronary atherosclerosis. It has been demonstrated that the annual rate of progression of coronary calcium load after heart transplantation is low [36]. The accurate selection of heart patient donors allows to obtain a group of patients with a low initial calcium content as in our study population, where 80% of HT patients had a value of CAC score less than 100. In the present investigation 50 out of 80 patients with CAC score < 100 did not show the presence of CAV and in this subgroup the AER was significantly higher in patients with reduced MPR as compared to those with normal MPR (17.5% vs. 6.5, *p* <.05). Thus, the complex interplay between epicardial and microvascular disease in the evolution of graft vasculopathy and events in the short and long-term follow-up should be better investigated in a larger patient’s selection of HT patients.

### Study limitation

There are limitations in this study that merit discussion. First, this is a single-center, observational study where patients did not have a standardized protocol to send patients to invasive angiography in their follow-up. Moreover, the mean time between ICA or CCTA and the PET/CT scan is not close, and it is variable among these patients to better evaluate the correlation of the imaging findings.

Second, patient selection is not large enough to understand all the possible combinations to risk stratify according to the different variables obtained by PET/CT imaging.

Moreover, average interval between HT and PET study is very long (13 years) with a follow-up interval of 2 years. Many of the high-risk patients might have already had cardiac events/death before the PET study while these enrolled patients might have had relatively better prognostic profiles.

## Conclusion

In HT patients both the presence of CAV and reduced MPR was associated with a poor prognosis and higher risk of adverse events. Interestingly, in patients without CAV the presence of reduced MPR was associated with higher risk and lower event-free survival as compared to patients with normal MPR, suggesting that the early noninvasive evaluation of microcirculatory dysfunction in HT recipients would provide better risk stratification and subsequent modification of treatment strategies.

## Data Availability

The datasets generated during and/or analysed during the current study are available from the corresponding author on reasonable request.

## Abbreviations

CAC: Coronary artery calcium
CAD: Coronary artery disease
CAV: Cardiac allograft vasculopathy
CT: Computed Tomography
EAT: Epicardial adipose tissue
HT: Heart transplantation
LVEF: Left ventricle ejection fraction
MPR: Myocardial perfusion reserve
MBF: Myocardial blood flow
PET: Positron emission tomography
TPD: Total perfusion defect

## Declarations

### Informed approval

Informed consent was obtained from all individual participants included in the study.
